# Geoeffective interplanetary magnetic field (IMF) from in situ data: realistic versus idealized spiral IMF

**DOI:** 10.1038/s41598-023-36499-1

**Published:** 2023-06-14

**Authors:** Mario Bandić, Giuliana Verbanac, Slaviša Živković

**Affiliations:** 1 Astronomical Observatory Zagreb, Zagreb, Croatia; 2 Department of Geophysics, Faculty of Science, University of Zagreb, Zagreb, Croatia; 3 State School Franjo Hanaman, Drenovci, Croatia

**Keywords:** Magnetospheric physics, Solar physics

## Abstract

The geoeffective, southward IMF ($B_\text {s}$) given in the GSM reference frame as nature presents is compared with that based on idealized, spiral IMF. We obtained $B_\text {s}$ and $B_\text {s}$ sorted by the IMF polarity ($B_\text {s}$ fields) from in situ data at a high 16-second resolution. Idealized IMF is derived by omitting the fluctuation of the IMF in the GSEQ Z-direction. Results are: the absolute value of realistic $B_\text {s}$ is larger than the one from idealized IMF; realistic $B_\text {s}$ polarity fields exist in all seasons, while those from idealized IMF exist only around spring/fall when the IMF points toward/away from the Sun; idealized $B_\text {s}$ fields match the predictions of the Russell–McPherron (RM) model almost ideally. The present study has resolved the issue related to the patterns and absolute values of the observed $B_\text {s}$ fields and those from the RM model that assumes an idealized IMF. It confirms that $B_{z,\text {GSEQ}}$ plays a crucial role for $B_\text {s}$. Finally, it paves a way to properly link the variations seen in geomagnetic activity with the pattern of the measured $B_\text {s}$ fields.

## Introduction

Interplanetary magnetic field (IMF) is the extension of the coronal magnetic field dragged by the solar wind to the interplanetary space. The open field lines coming from the opposite sides of the dipole equator represent magnetic fields of different IMF polarity: toward the Sun in one hemisphere and away from the Sun in the other hemisphere (depending on the solar cycle). Because of the rotation of the Sun, IMF becomes twisted into an Archimedean spiral (Parker spiral) in the solar equatorial plane^1,2^. The southward component of IMF given in the GSM reference frame, $B_\text {s}$, has for a long time been considered as a quantity that is efficient in the transfer of particles and energy from the solar wind into the magnetosphere^3–9^. For studying seasonal variations in magnetospheric activity related to $B_\text {s}$, it is important to know the pattern of $B_\text {s}$.

Observed $B_\text {s}$ and $B_\text {s}$ separated according to the IMF polarity were analyzed in detail in the recent study by^10^ (thereafter V.B. 2021). They demonstrated that $B_\text {s}$ sorted by the IMF polarity (thereafter polarity fields) can both exist in all seasons. In unfavorable seasons, that are fall/spring when the IMF points toward/away from the Sun, the field values are lowered, but they are not zero. This confirms that the patterns of the polarity fields are not in full accordance with the predictions of Russell-McPherron^11^ (thereafter RM) model, nor are their absolute values. Recall that RM provided a simplified model of $B_\text {s}$ assuming an idealized situation in which the constant IMF field lies exactly along the spiral angle and is equally likely toward or away from the Sun throughout the year. According to this model the polarity fields are zero in unfavorable seasons. The forms of the pattern without absolute values of the observed $B_\text {s}$ is given in^12^ (their Figure 2d) and of observed polarity fields in^13^ (their Figure A3). According to the first study^12^, observed $B_\text {s}$ agrees with the RM pattern, but its absolute value is not provided. The second mentioned study^13^, demonstrated that the observed polarity fields show the “pair of spectacles” pattern, and that on the other hand those predicted from the RM model are zero in fall/spring for toward/away from the Sun field (unfavorable seasons). These results support findings of V.B. 2021. Recently^14^ commented that the patterns of the observed $B_\text {s}$ fields obtained in V.B. 2021 are not the right ones, but indeed that predicted by the RM model of $B_\text {s}$. In^15^ we have provided arguments and explanations why we disagree with this comment. Further, in the study^16^ we have obtained $B_\text {s}$ fields at the high 16-second resolution and have shown that regardless of the resolution, $B_\text {s}$ fields exist in all seasons, also in unfavorable seasons.

It is clear, at least from our point of view, that the conclusions related to $B_\text {s}$ fields should be based on observations, and not on the model which does not take into account the realistic features of IMF. Namely, the magnetic field deviations from the Parker spiral direction have been reported by many authors (e.g.^17–24^) and are confirmed with recent Parker Solar Probe observations (e.g.^25,26^ and references therein). Some previous studies pointed out that IMF fluctuation about the spiral angle cannot be ignored in studies of semiannual, annual variations in geomagnetic activity (e.g.^27–30^) and in study of $B_\text {s}$ fields (V.B. 2021). Thus, the deviations from the Parker spiral are an observable feature of the IMF which affect the solar wind coupling with the magnetosphere through $B_\text {s}$ and other coupling functions and should not be simply neglected when studying the relationship between $B_\text {s}$ and magnetospheric quantities.

Nevertheless, to avoid that it further remains unexplained why the patterns and absolute values of the observed polarity fields and the absolute value of $B_\text {s}$ are not in line with the prediction of the RM model of $B_\text {s}$, the present study aims to answer the following questions: (a) since the RM model does not describe the observations, is there any data set that the model can match, (b) if the latter is the case, what characteristics does that data set have?

Besides providing explicit evidence and explanation of the observed discrepancies between observed $B_\text {s}$ fields and RM model prediction, this study contributes to properly connect the variations seen in magnetospheric activity with the observed, measured $B_\text {s}$ fields.

The paper is organized as follows. The next section is devoted to data and method. In “Results” we present the obtained results. Characteristics of $B_\text {s}$ fields are presented in “Characteristics of *B*_s_ fields: complete versus incomplete fields”. Then follow the discussion and conclusion.

## Data and method

In this paper for the period 1998–2017 we used IMF components measured by Magnetometer (MAG^31^) onboard the Advanced Composition Explorer (ACE^32^) satellite, given in GSM at high 16-second resolution. First, we derive observed $B_\text {s}$ fields (thereafter complete observed fields) from $B_{z,\text {GSM}}$ and $B_{y,\text {GSEQ}}$ that we rectified at 16-seconds. The fields are defined as: $B_\text {s}$=$B_{z,\text {GSM}}<0$ and undefined otherwise, $B_\text {s}$=$B_\text {s}({\scriptstyle B_{y,\text {GSEQ}}} < 0)$ for IMF pointing toward the Sun and $B_\text {s}$=$B_\text {s}({\scriptstyle B_{y,\text {GSEQ}}} > 0)$ for IMF pointing away from the Sun, respectively. In the next step, the IMF components given in GSM are transformed to GSEQ. Following the assumption that RM made to obtain their model, for each IMF vector we set $B_{z,\text {GSEQ}}$ to zero. In such a way obtained IMF vectors which have all components projected to the X-Y GSEQ plane are transformed back to GSM.

**Figure 1:**
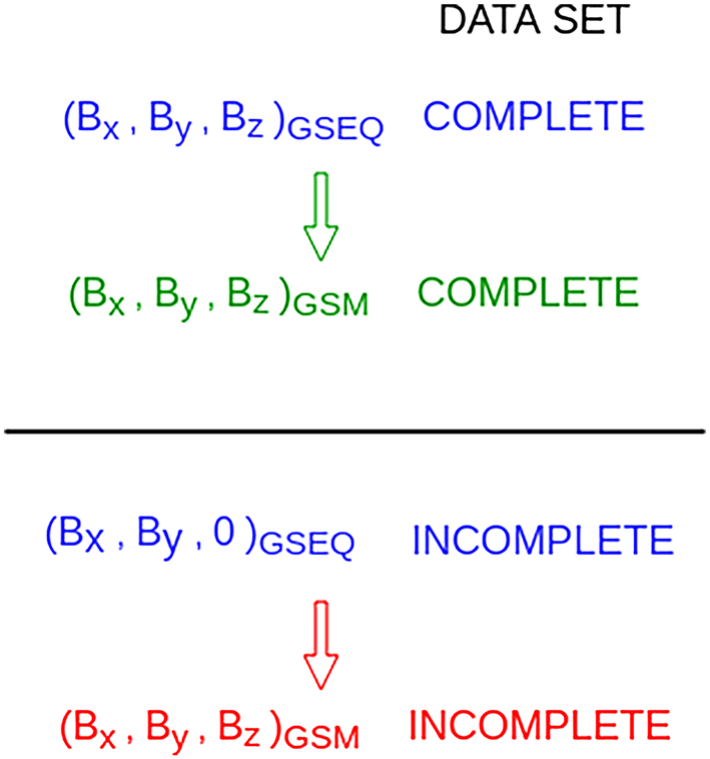
Schematic illustrating differences between complete and incomplete IMF vectors in GSEQ (marked in blue) and in GSM (marked in green for the complete data set and in red for the incomplete data set).

Generally, we have:

1 $$\begin{aligned} B_{z,\scriptscriptstyle {\text {GSM}}} = \underbrace{ B_{y,\scriptscriptstyle {\text {GSEQ}}} \, \sin \alpha }_{\text {I}} + \underbrace{ B_{z,\scriptscriptstyle {\text {GSEQ}}} \, \cos \alpha }_{\text {II}} \end{aligned}$$

so only the first term (denoted as I) in expression (1) remains. From such $B_{z,\text {GSM}}$ data set we derive $B_\text {s}$ fields, thereafter called incomplete observed $B_\text {s}$ fields. The angle $\alpha$ is the rotation angle between the GSEQ and GSM frames. Differences between complete and incomplete IMF vectors are schematically presented in Fig. 1. Complete and incomplete data sets in GSEQ (marked in blue) have the same X and Y components, but different Z components. By transforming from GSEQ to GSM, different IMF vectors of complete and incomplete data sets are obtained (marked in green and red).

To show both complete and incomplete observed $B_\text {s}$ fields as a function of day of the year (DOY), we calculate their means by averaging all 16-second values of all studied years within DOY intervals of 14-day and 1-month. To display data as pictograms in DOY-UT representation, we calculated means by averaging all 16-second values within the UT-interval of an hour for all days within the chosen DOY interval for all years. Further, for discussion purposes, we derived the hour-of-year means (365$\times$24 values within a year, thereafter HOY averages) by averaging all 16-second values of the same hours of all 20 considered years. These data are shown in the DOY-UT representation of 1-day $\times$ 1-hour. By averaging $B_\text {s}$ fields over many years (here 20 years), we reduced them to the epoch of 1 year. In the following they will be denoted as $\left\langle B_\text {s} \right\rangle$, $\left\langle B_\text {s}({\scriptstyle B_{y,\text {GSEQ}}} < 0) \right\rangle$ and $\left\langle B_\text {s}({\scriptstyle B_{y,\text {GSEQ}}} > 0) \right\rangle$. We derive also the final means of $B_\text {s}$ fields by averaging all 16-second values within 20 years, named fin averages ($\left\langle B_\text {s} \right\rangle _{\text {fin}}$, $\left\langle B_\text {s}({\scriptstyle B_{y,\text {GSEQ}}} < 0) \right\rangle _{\text {fin}}$, $\left\langle B_\text {s}({\scriptstyle B_{y,\text {GSEQ}}} > 0) \right\rangle _{\text {fin}}$).

Finally, we obtain $B_\text {s}$ fields from the RM model following details about their calculations provided in V.B. 2021 which are based on assumptions made by RM. Let us briefly recall that according to the RM approach the constant IMF lies along the spiral angle, thus $B_{z,\text {GSEQ}}$ = 0. They calculated $\left\langle B_\text {s}({\scriptstyle B_{y,\text {GSEQ}}} < 0) \right\rangle$ and $\left\langle B_\text {s}({\scriptstyle B_{y,\text {GSEQ}}} > 0) \right\rangle$. The $\left\langle B_\text {s} \right\rangle$ was not derived directly, but as the average of the two polarity fields.

The polarity fields are calculated as follows (according to expression 6 given in Appendix of V.B. 2021):

2 $$\begin{aligned} \begin{aligned} \left\langle B_\text {s}({\scriptstyle B_{y,\text {GSEQ}}}< 0) \right\rangle= & \: {} \left\langle B_{y,{\text {GSEQ}}}< 0 \right\rangle _{\text {fin}} \sin \alpha , \quad \alpha> 0{} & {} {} & {} \\ \left\langle B_\text {s}({\scriptstyle B_{y,\text {GSEQ}}}> 0) \right\rangle= & \: {} \left\langle B_{y,{\text {GSEQ}}} > 0 \right\rangle _{\text {fin}} \sin \alpha , \quad \alpha < 0{} & {} .{} & {} \\ \end{aligned} \end{aligned}$$

RM set $\left\langle B_{y,{\text {GSEQ}}} < 0 \right\rangle _{\text {fin}}$ and $\left\langle B_{y,{\text {GSEQ}}} > 0 \right\rangle _{\text {fin}}$, which represent means over a long time span, to -5/$\sqrt{2}$ and 5/$\sqrt{2}$, respectively. In the present study to obtain the polarity fields using the above formula, we calculate $\left\langle B_{y,{\text {GSEQ}}} < 0 \right\rangle _{\text {fin}}$ and $\left\langle B_{y,{\text {GSEQ}}} > 0 \right\rangle _{\text {fin}}$ by averaging all corresponding 16-second $B_{y,{\text {GSEQ}}} < 0$ and $B_{y,{\text {GSEQ}}} > 0$ values within the considered time span (1998-2017). The obtained value of ± 3.40 nT was used to predict polarity fields. In the same way we calculate also the $\left\langle B_{z,{\text {GSEQ}}} < 0 \right\rangle _{\text {fin}}$ and $\left\langle B_{z,{\text {GSEQ}}} > 0 \right\rangle _{\text {fin}}$ averages and the obtained value of ± 2.45 nT will be used for discussion of the results.

RM postulated $\left\langle B_\text {s} \right\rangle$ to be:

3 $$\begin{aligned} \left\langle B_s \right\rangle = \frac{1}{2} \, \Bigl ( \left\langle B_\text {s}({\scriptstyle B_{y,\text {GSEQ}}} < 0) \right\rangle + \left\langle B_\text {s}({\scriptstyle B_{y,\text {GSEQ}}} > 0) \right\rangle \Bigr ). \end{aligned}$$

Since $\left\langle B_\text {s}({\scriptstyle B_{y,\text {GSEQ}}} < 0) \right\rangle$ and $\left\langle B_\text {s}({\scriptstyle B_{y,\text {GSEQ}}} > 0) \right\rangle$ do not overlap for any $\alpha$, as commented in V.B. 2021, the above formula turns to:

4 $$\begin{aligned} \left\langle B_s \right\rangle = \frac{1}{2} {\left\{ \begin{array}{ll} \left\langle B_\text {s}({\scriptstyle B_{y,\text {GSEQ}}}< 0) \right\rangle , \quad \alpha> 0 \\ \left\langle B_\text {s}({\scriptstyle B_{y,\text {GSEQ}}} > 0) \right\rangle , \quad \alpha < 0 \\ \end{array}\right. } \end{aligned}$$

which we assign as $\left\langle B_\text {s} \right\rangle$ from the RM model.

The obtained characteristics of $\left\langle B_\text {s} \right\rangle$ fields from complete and incomplete datasets, discussed in section “Characteristics of *B*_s_ fields: complete versus incomplete fields” , have indicated how $\left\langle B_\text {s} \right\rangle$ has to be expressed as function of the two polarity fields. It is shown that factor 1/2 in expression (4) has to be changed to 1 and that $\left\langle B_\text {s} \right\rangle$ from the RM model shall be calculated using expression (8). $\left\langle B_\text {s} \right\rangle$ derived using formula (8) will be denoted as $\left\langle B_\text {s} \right\rangle$ predicted from the corrected RM model. Note that the temporal behavior of $B_\text {s}$ fields depends on angle $\alpha$ and is not affected by the initial IMF resolution. Since $\alpha$ exhibits annual and diurnal variations, we used hourly values of $\alpha$ which we find to be sufficient.

## Results

**Figure 2:**
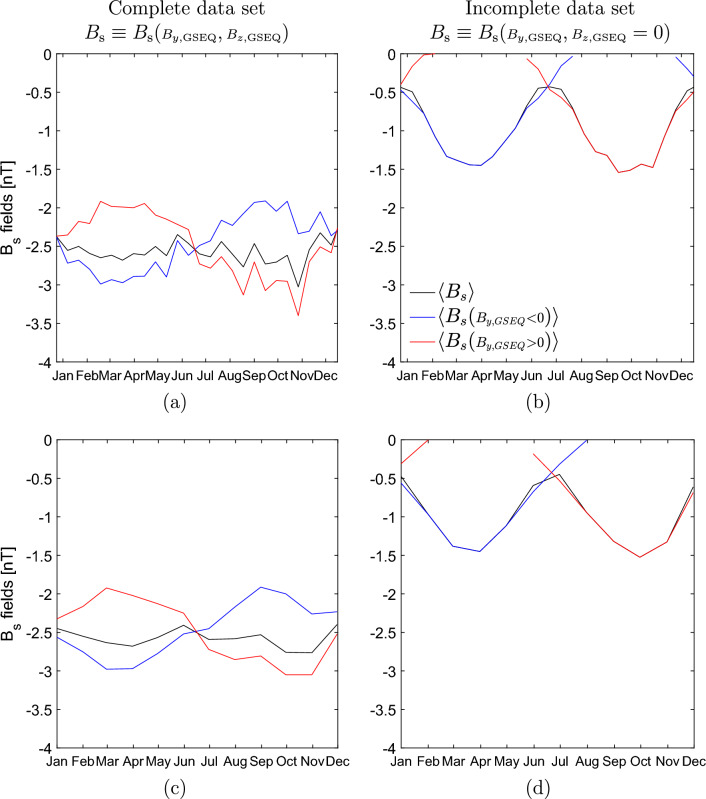
Complete (**a,c**) and incomplete (**b,d**) observed $B_\text {s}$ fields averaged on DOY-interval of 14-day (first row) and 1-month (second row). In all figure panels, $\left\langle B_\text {s} \right\rangle$, $\left\langle B_\text {s}({\scriptstyle B_{y,\text {GSEQ}}} < 0) \right\rangle$ and $\left\langle B_\text {s}({\scriptstyle B_{y,\text {GSEQ}}} > 0) \right\rangle$ are depicted in black, blue and red, respectively.

Figure 2a,c depicts complete and Fig. 2b,d incomplete observed $B_\text {s}$ fields averaged on DOY-interval of 14-day and 1-month. The complete polarity fields exhibit the “pair of spectacles” pattern. They show enhancements in the favorable and reductions in unfavorable seasons of approximately the same amplitude. Amplitudes of complete $\left\langle B_\text {s} \right\rangle$ are smaller than amplitudes that the complete polarity fields attain in their favorable seasons (black line in Fig. 2a,c is above the blue/red line in spring/fall). The $B_\text {s}$ fields oscillate around the average value (fin average) that for all of them amounts to $\sim -2.6$ nT. On the other hand, the incomplete observed polarity fields do not exhibit the “pair of spectacles” pattern. Each of them lacks part of the pattern in unfavorable seasons: there is no $\left\langle B_\text {s}({\scriptstyle B_{y,\text {GSEQ}}} < 0) \right\rangle$ in fall and no $\left\langle B_\text {s}({\scriptstyle B_{y,\text {GSEQ}}} > 0) \right\rangle$ in spring. The fields oscillate around $\sim -0.5$ nT, the value that is about five times smaller than the average about which the complete observed polarity fields oscillate. Also, their amplitudes are about twice the value of the amplitudes of the complete observed fields. The incomplete $\left\langle B_\text {s} \right\rangle$ in spring and fall matches the values of $\left\langle B_\text {s}({\scriptstyle B_{y,\text {GSEQ}}} < 0) \right\rangle$ and $\left\langle B_\text {s}({\scriptstyle B_{y,\text {GSEQ}}} > 0) \right\rangle$, respectively. Note that these results hold regardless of the resolution at which the fields are presented.

**Figure 3:**
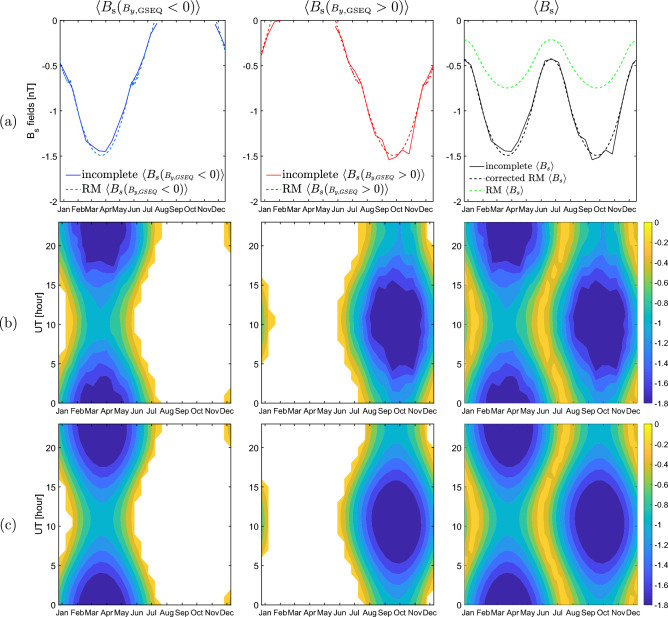
(**a**) Solid lines: incomplete $B_\text {s}$ fields at 14-day time resolution (repeated from Fig. 2b), dashed lines (blue, red, green): hourly values of RM model predictions and of $\left\langle B_\text {s} \right\rangle$ from corrected RM model obtained using formula (8) (black dashed line), (**b**) contour plots of incomplete observed fields (**c**) contour plots of the polarity fields from RM model and that of $\left\langle B_\text {s} \right\rangle$ from the corrected RM model. All contour plots are shown on DOY-UT representation of 14-day $\times$ 1-hour.

Figure 3 shows: (a) incomplete observed fields along with the predictions of the RM model (calculated using expression (2) and expression (4)). Additionally, $\left\langle B_\text {s} \right\rangle$ predicted from the corrected RM model (expression (8)) is depicted. (b) The contour plots of incomplete observed fields and (c) contour plots of the polarity fields from the RM model and that of $\left\langle B_\text {s} \right\rangle$ from the corrected RM model. Plots related to the polarity fields show that they are in very good agreement with the predictions of the RM model. Both the patterns and the absolute values are in accordance. The $\left\langle B_\text {s}({\scriptstyle B_{y,\text {GSEQ}}} < 0) \right\rangle$ exists only around spring and $\left\langle B_\text {s}({\scriptstyle B_{y,\text {GSEQ}}} > 0) \right\rangle$ only around fall. Amplitude and absolute value of incomplete observed $\left\langle B_\text {s} \right\rangle$ is not in accordance with $\left\langle B_\text {s} \right\rangle$ predicted by the RM model, but matches well $\left\langle B_\text {s} \right\rangle$ calculated using expression (8).

**Figure 4:**
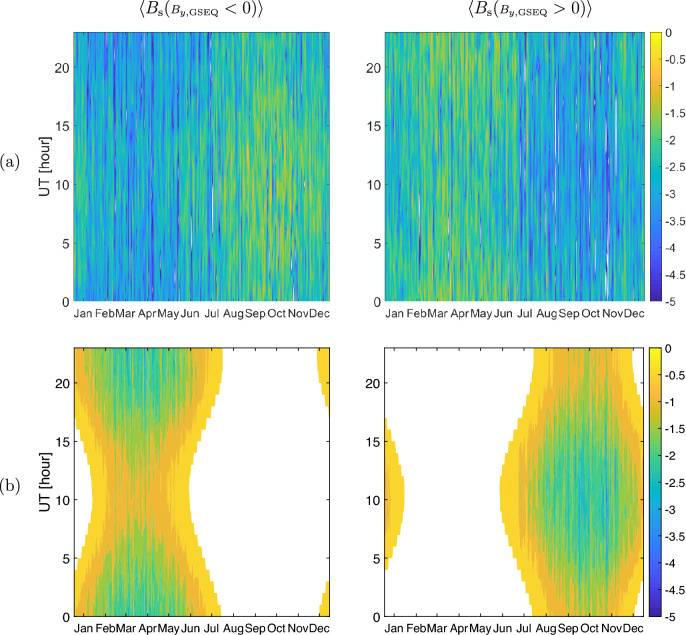
Contour plots of HOY averages: (**a**) complete and (**b**) incomplete polarity fields on DOY-UT representation of 1-day $\times$ 1-hour.

Figure 4 shows the contour plots of the complete and incomplete polarity fields defined on the HOY scale.

## Characteristics of $B_\text {s}$ fields: complete versus incomplete fields

In this section we focus on the existence of $\left\langle B_\text {s}({\scriptstyle B_{y,\text {GSEQ}}} < 0) \right\rangle$ and $\left\langle B_\text {s}({\scriptstyle B_{y,\text {GSEQ}}} > 0) \right\rangle$ within the year, explanation of the observed features and on the relationship between $B_\text {s}$ fields.

### Complete $B_\text {s}$ fields

According to Fig. 2a,c complete $\left\langle B_\text {s}({\scriptstyle B_{y,\text {GSEQ}}} < 0) \right\rangle$ and $\left\langle B_\text {s}({\scriptstyle B_{y,\text {GSEQ}}} > 0) \right\rangle$ can exist at the same point in time. Figure 4a confirms that this is valid for every HOY and that the results are not influenced by averaging the 16-second field values on DOY interval of 14-day and 1-month. As noted in V.B. 2021, the $B_\text {s}$ will exist at some point in time as long as the following condition is satisfied:

5 $$\begin{aligned} B_{z,{\text {GSEQ}}} < - B_{y,{\text {GSEQ}}} \tan \alpha \end{aligned}$$

Since the signs of $B_{y,\text {GSEQ}}$ and $B_{z,\text {GSEQ}}$ vary randomly through the years and thus they are not seasonal dependent, on averaging over many years both complete $\left\langle B_\text {s}({\scriptstyle B_{y,\text {GSEQ}}} < 0) \right\rangle$ and $\left\langle B_\text {s}({\scriptstyle B_{y,\text {GSEQ}}} > 0) \right\rangle$ fields exist in all seasons (favorable and in unfavorable seasons). This explains why these fields can exist at any HOY within the year (as observed in Fig. 4a).

Further, from Fig. 2a,c it follows that $\left\langle B_\text {s} \right\rangle$ is not a simple average of $\left\langle B_\text {s}({\scriptstyle B_{y,\text {GSEQ}}} < 0) \right\rangle$ and $\left\langle B_\text {s}({\scriptstyle B_{y,\text {GSEQ}}} > 0) \right\rangle$, but can be expressed as a function of the two fields as follows:

6 $$\begin{aligned} \left\langle B_\text {s} \right\rangle = f_1(\alpha ) \left\langle B_\text {s}({\scriptstyle B_{y,\text {GSEQ}}} < 0) \right\rangle + f_2(\alpha ) \left\langle B_\text {s}({\scriptstyle B_{y,\text {GSEQ}}} > 0) \right\rangle . \end{aligned}$$

The seasonal variations is contained in $f_1$ and $f_2$, and are that of $\alpha$. The functions $f_1(\alpha )$ and $f_2(\alpha )$ are such that give more weight to $\left\langle B_\text {s} \right\rangle$ in the favorable seasons of the polarity fields. If $\left\langle B_\text {s} \right\rangle$ were the average of the two polarity fields then it would attain a nearly constant value (that of fin average) and would not show the semiannual variation.

### Incomplete $B_\text {s}$ fields

Fig. 3a shows that incomplete observed $\left\langle B_\text {s}({\scriptstyle B_{y,\text {GSEQ}}} < 0) \right\rangle$ exists around spring and $\left\langle B_\text {s}({\scriptstyle B_{y,\text {GSEQ}}} > 0) \right\rangle$ around fall. In summer and winter these fields overlap. Figure 3b suggests that these are independent on UT in fall and spring, but dependent on UT in summer and winter. Thus, there is an indication that the fields in all seasons do not exist at the same point in time. This issue solves Fig. 4b by clearly revealing that at each single HOY the field is either $\left\langle B_\text {s}({\scriptstyle B_{y,\text {GSEQ}}} < 0) \right\rangle$ or $\left\langle B_\text {s}({\scriptstyle B_{y,\text {GSEQ}}} > 0) \right\rangle$ regardless of the season. In this way we have shown that the overlap of the polarity fields in summer and winter seen in Fig. 3a is caused by their averaging on DOY-interval of 14-day which does not enable to resolve the UT dependence.

Adopting the assumption that $B_{z,\text {GSEQ}}$ equals zero to obtain incomplete fields, the expression (5) reduces to:

7 $$\begin{aligned} \begin{aligned} B_{y,\text {GSEQ}}<0{} & \quad {} \text {for} \quad \alpha> 0{} & {} {} & {} \text {and} \\ B_{y,\text {GSEQ}} >0{} & \quad {} \text {for} \quad \alpha < 0{} & {} {} & {} \end{aligned} \end{aligned}$$

implying that at some point in time $B_\text {s}$ can be either $B_\text {s}({\scriptstyle B_{y,\text {GSEQ}}} < 0)$ or $B_\text {s}({\scriptstyle B_{y,\text {GSEQ}}} > 0)$. This explains why the incomplete observed $\left\langle B_\text {s}({\scriptstyle B_{y,\text {GSEQ}}} < 0) \right\rangle$ and $\left\langle B_\text {s}({\scriptstyle B_{y,\text {GSEQ}}} > 0) \right\rangle$ are mutually exclusive for each HOY, as Fig. 4b shows. That indicates that $f_1$ and $f_2$ are constants (equal to 1) in this case and that expression (6) turns to:

8 $$\begin{aligned} \left\langle B_s \right\rangle = {\left\{ \begin{array}{ll} \left\langle B_\text {s}({\scriptstyle B_{y,\text {GSEQ}}}<0) \right\rangle , \quad \alpha> 0 \\ \left\langle B_\text {s}({\scriptstyle B_{y,\text {GSEQ}}} >0) \right\rangle , \quad \alpha < 0. \\ \end{array}\right. } \end{aligned}$$

### RM model

$B_\text {s}$ fields both from the RM model and from the incomplete dataset are based on the same assumption that $B_{z,\text {GSEQ}}$ is zero. Because of that $B_\text {s}({\scriptstyle B_{y,\text {GSEQ}}} < 0)$ and $B_\text {s}({\scriptstyle B_{y,\text {GSEQ}}} > 0)$ predicted by the RM model (expression 2) have the same characteristics as incomplete polarity fields: they are mutually exclusive for each HOY. For the model to be consistent with the incomplete dataset this feature must be taken into account when calculating $\left\langle B_\text {s} \right\rangle$ from the polarity fields. Thus, $\left\langle B_\text {s} \right\rangle$ shall be calculated using expression (8), which represents the corrected RM model, instead of expression (4). This is clearly seen in Fig. 3a (third column): $\left\langle B_\text {s} \right\rangle$ from the corrected RM model (black dashed line) matches the incomplete observed $\left\langle B_\text {s} \right\rangle$ (black solid line), while $\left\langle B_\text {s} \right\rangle$ from the RM model (green dashed line) does not.

## Discussion

Results have shown that complete $B_\text {s}$ fields oscillate around the mean value (fin average) which is 5 times higher than the average about which the incomplete fields oscillate. We attribute that to the larger contribution of the second term in expression (1) to $B_\text {s}$. For the examined period (1998-2017), the fin average of $B_{y,\text {GSEQ}}$ sorted by IMF polarity (± 3.40 nT) is greater than the fin average of $B_{z,\text {GSEQ}}$ sorted by IMF polarity (± 2.45 nT), which would indicate a larger contribution of $B_{y,\text {GSEQ}}$ component to $B_\text {s}$ fields. But, since IMF that is ordered in GSEQ contributes to $B_{z,\text {GSM}}$ in combination with $\alpha$ which is in the range ± 37$^{\circ }$, the second term in expression (1) indeed dominates. The importance of the second term has already been noticed by^27^ and^28^ and discussed in more detail in V.B. 2021.

Further, we showed that the amplitude of the incomplete $\left\langle B_\text {s} \right\rangle$ is too large compared to the complete $\left\langle B_\text {s} \right\rangle$. With the corrected relationship between the $B_\text {s}$ fields (expression 8), compared to the one proposed by RM, the absolute value of the incomplete field is matched. Nevertheless even this value, nor the one postulated by RM, is not in accordance with observations. Since for the incomplete observed $B_\text {s}$ fields each HOY in all seasons is characterized with one of the two polarities, it follows that at some HOY in all 20 years the polarity of the field is always the same. This further means that polarity in this idealized IMF situation is seasonal dependent. Further, the incomplete observed $B_\text {s}$ is obtained by merging both polarity fields (expression 8) rather than be an average of both (expression 3). This is caused by unreal polarity separation as explained above. In this context, in the present study we have made progress by deriving the general relationship between the fields (expression 6) which shows that $B_\text {s}$ can not be expressed as a simple average of $B_\text {s}$ ordered by IMF polarity, as assumed by RM. The analysis and conclusions are based on realistic situation in which for specific HOY, the fields can randomly be of any polarity (toward or away from the Sun). Therefore, by averaging over many years (here 20 years) both $\left\langle B_\text {s}({\scriptstyle B_{y,\text {GSEQ}}} < 0) \right\rangle$ and $\left\langle B_\text {s}({\scriptstyle B_{y,\text {GSEQ}}} > 0) \right\rangle$ can appear in favorable and in unfavorable seasons. The averaging retains the information about the existence of both field polarity.

Crooker and Siscoe^33^ already in 1986 pointed out (on pages 209–210): “... although the polarity effect itself is an outstanding feature in data sets separated according to polarity, the net effect of mixed polarities makes only a small contribution to the semiannual variation. When a realistic distribution of the north–south component of the IMF is used in a model of the polarity effect, the annual variation of geomagnetic activity for a given polarity is not at nearly zero level for half of the year, as it would be for an idealized spiral IMF, but instead varies gradually in a sinusoidal-like way. Consequently, the net effect of these two annual variations of opposite phase is a semiannual wave of amplitude considerably smaller than that predicted on the basis of an idealized spiral IMF”. These very advanced notices that are in line with results obtained in the present study have been unfortunately forgotten and not considered. In our view, probably because in the interpretation of semiannual, annual and diurnal variations of magnetospheric quantities, the observed $B_\text {s}$ fields have not been considered and the RM model based on idealized spiral IMF was adopted. Consequently, what has been shown by us here that the semiannual amplitude of realistic (observed) $B_\text {s}$ is small, and much smaller than the one from the RM model, along with confirmation that the pattern of polarity fields is the “pair of spectacles” pattern (two annual sinusoidal-like variations of opposite phase) should really be taken into account when $B_\text {s}$ is considered to be the causative agent of geomagnetic activity.

The present study has clearly shown why the RM model based on the idealized IMF cannot match the observed fields by providing the data set (incomplete data set) that this model accommodates. The discrepancy between the $B_\text {s}$ from complete and from incomplete data sets becomes especially noticeable in the cases when the polarities are considered separately.

Note that we did not analyze variations in any magnetospheric quantity. Nevertheless, based on the obtained results, in the following we provide some possible explanations why studies that considered different mechanisms responsible for variations in geomagnetic activity commented that the contribution of $B_\text {s}$ is small (e.g.^34^). First, if noticed that $B_\text {s}$ has little influence on the semiannual variation it may not necessarily be because $B_\text {s}$ is not important in confront to other effects, but just because the amplitude of the semiannual variation of observed $B_\text {s}$ is low. Further, if one finds in magnetospheric quantity separated according to IMF polarity an enhancement in the favorable season and reduction in the unfavorable season, but not zero activity in unfavorable seasons, it could be a sign of the influence of polarity fields. Then, this indicates that $B_\text {s}$ fields do contribute to the variations seen in magnetospheric quantity. In particular when geomagnetic indices are sorted by IMF polarity, the impact of the complete polarity fields, and not the impact of incomplete ones which the RM model well described, becomes clearly evident. For instance^27,28^ and^35^ obtained enhancements in the favorable and reductions in unfavorable seasons (a pattern similar to the “pair of spectacles” pattern) when geomagnetic indices AL, am and AE, and am are ordered by the IMF polarity respectively. The obtained variations in these geomagnetic indices reveal the pattern of the complete $B_\text {s}$ fields shown in our Fig. 2a,c. Further, a recent study by^36^ has shown how important it is to use the complete, observed pattern of $B_\text {s}$ as input when modeling geomagnetic indices Dst and Kp sorted by IMF polarity. This work used the information from coronal holes on the Sun that are of a strictly defined polarity. As the prior function (input for the model) they employed the sinusoidal function, the form of a realistic $B_\text {s}$ polarity field. In this way the seasonal variations in the geomagnetic activity were well reproduced. If as a prior function the patterns of incomplete $B_\text {s}$ polarity fields were chosen, then the method would not lead to meaningful results.

There are solar wind-magnetosphere coupling functions which are combinations of different measured interplanetary parameters (for details about different coupling functions the reader is referred to the study by^37^). They are used to quantitatively predict magnetospheric activity. Most of them contain IMF orientation factor F($\theta$) via sin($\theta$/2) on some exponents, where $\theta$ is the clock angle defined as $\tan (\theta$) = $|B_y|/B_z$ in GSM. These coupling functions sorted by the IMF polarity are not zero in unfavorable seasons. They exhibit enhancements and reductions within the year (e.g. see Figure 12b in^35^), similar to the pattern of observed, complete $B_\text {s}$ polarity fields. The reason for that is that sin ($\theta$/2) allows stronger coupling during southward and weaker coupling during northward pointing IMF $B_{z,\text {GSM}}$ component. This indicates that much of the IMF dependence reflected in geomagnetic activity originates from the southward component of the IMF given in GSM, further confirming the importance to clarify the real, observed pattern of $B_\text {s}$ fields.

To summarize, in light of the obtained results and the above discussion, the observed $B_\text {s}$ fields are those that can contribute to the magnetospheric activity and not the incomplete fields. We note that discussion related to the imprint of $B_\text {s}$ fields in geomagnetic quantities does not rule out other parameters and mechanisms that besides $B_\text {s}$ can affect seasonal variations in geomagnetic activity.

## Conclusion

Although recent studies have already provided evidence that the RM model of $B_\text {s}$ does not match the observations, the present work has explicitly proved that and has provided explanations. We have derived incomplete observed $B_\text {s}$ fields and have demonstrated that it is exactly this data set that the RM model can describe. Comparison of the $B_\text {s}$ fields obtained from the observed data set, incomplete observed data set and those predicted with the RM model allows us to explicitly deduce where the differences between the observations and model predictions come from. The results have confirmed that $B_{z,\text {GSEQ}}$ plays a significant role and in combination with angle $\alpha$ it becomes crucial to obtain $B_\text {s}$ fields as nature presents. In summary, the present study has resolved the issue related to the pattern and absolute value of the observed $B_\text {s}$ fields and those obtained with the RM model. The results have pointed out that it is very important to consider the pattern of observed $B_\text {s}$ fields when interpreting semiannual (annual) variations in magnetospheric quantities and moreover when modeling geomagnetic indices. Finally, it has shown that the new model of the $B_\text {s}$ fields which will take into account the fluctuation of IMF about the spiral direction, the most probable IMF orientation, and in that way be in accordance with observations is needed. This is the subject of our work in progress.

## Data Availability

The interplanetary magnetic field data analysed during the current study are available at https://izw1.caltech.edu/ACE/ASC/level2/lvl2DATA_MAG.html.

## References

[1] Parker EN (1958). Dynamics of the interplanetary gas and magnetic fields. Astrophys. J..

[2] Ness NF, Wilcox JM (1964). Solar origin of the interplanetary magnetic field. Phys. Rev. Lett..

[3] Fairfield DH, Cahill LJ (1966). Transition region magnetic field and polar magnetic disturbances. J. Geophys. Res..

[4] Gonzalez WD, Tsurutani BT (1987). Criteria of interplanetary parameters causing intense magnetic storms ( D$$_{st}$$$$<$$ -100 nT). Planet. Sp. Sci..

[5] Gonzalez WD (1994). What is geomagnetic storms. J. Geophys. Res..

[6] Gonzalez, W. D., Tsurutani, B. T. & Clúa de Gonzalez, A. L. Interplanetary origin of geomagnetic storms. *Sp. Sci. Rev.* **88**, 529–562. 10.1023/A:1005160129098 (1999).

[7] Zhang J, Dere KP, Howard RA, Bothmer V (2003). Identification of solar sources of major geomagnetic storms between 1996 and 2000. Astrophys. J..

[8] Gopalswamy N (2008). Solar connections of geoeffective magnetic structures. J. Atmos. Solar-Terrestrial Phys..

[9] Tsurutani BT, Lakhina GS, Hajra R (2020). The physics of space weather/solar-terrestrial physics (STP): What we know now and what the current and future challenges are. Nonlinear Process. Geophys..

[10] Verbanac G, Bandić M (2021). Origin and characteristics of the southward component of the interplanetary magnetic field. Solar Phys..

[11] Russell C, McPherron R (1973). Semiannual variation of geomagnetic activity. J. Geophys. Res..

[12] Lockwood M (2016). On the origins and timescales of geoeffective IMF. Sp. Weather.

[13] Lockwood, M. *et al.* Semi-annual, annual and Universal Time variations in the magnetosphere and in geomagnetic activity: 4. Polar Cap motions and origins of the Universal Time effect. *J. Sp. Weather Sp. Clim.* **11**, 15. 10.1051/swsc/2020077 (2021).

[14] Russell C (2022). Comment on: “Origin and characteristics of the southward component of the interplanetary magnetic field” by G. Verbanac and M. Bandić. Solar Phys..

[15] Verbanac, G. & Bandić, M. Reply to Comment on “Origin and characteristics of the southward component of the interplanetary magnetic field” by C.T. Russell. *Solar Phys.* **297**, 156. 10.1007/s11207-022-02086-2 (2022).

[16] Živković, S., Verbanac, G. & Bandić, M. Does the time resolution of the geoeffective IMF component influence its annual, semiannual and diurnal patterns? (submitted) (2023).

[17] Ness NF, Wilcox JM (1966). Extension of the photospheric magnetic field into interplanetary space. Astrophys. J..

[18] Hirshberg J, Colburn DS (1969). Interplanetary field and geomagnetic variations—A unifield view. Planet. Sp. Sci..

[19] Forsyth RJ, Balogh A, Smith EJ, Erdös G, McComas DJ (1996). The underlying Parker spiral structure in the Ulysses magnetic field observations, 1990–1994. J. Geophys. Res..

[20] Fisk LA (1996). Motion of the footpoints of heliospheric magnetic field lines at the Sun: Implications for recurrent energetic particle events at high heliographic latitudes. J. Geophys. Res..

[21] Burlaga LF, Ness NF (1997). Global patterns of heliospheric magnetic field polarities and elevation angles: 1990 through 1995. J. Geophys. Res..

[22] Fisk LA (2001). On the global structure of the heliospheric magnetic field. J. Geophys. Res..

[23] Borovsky, J. E. On the variations of the solar wind magnetic field about the Parker spiral direction. *J. Geophys. Res. (Space Physics)* **115**, A09101. 10.1029/2009JA015040 (2010).

[24] Borovsky JE (2021). Exploring the properties of the electron Strahl at 1 AU as an indicator of the quality of the magnetic connection between the Earth and the Sun. Front. Astron. Sp. Sci..

[25] Schwadron, N. A. & McComas, D. J. Switchbacks explained: Super-Parker fields—The other side of the sub-Parker spiral. *Astrophys. J.* **909**, 95. 10.3847/1538-4357/abd4e6. arXiv:2102.03696 (2021).

[26] Raouafi, N. E. *et al.* Parker solar probe: Four years of discoveries at solar cycle minimum. *Sp. Sci. Rev.* **219**, 8. 10.1007/s11214-023-00952-4. arXiv:2301.02727 (2023).

[27] Berthelier A (1976). Influence of the polarity of the interplanetary magnetic field on the annual and the diurnal variations of magnetic activity. J. Geophys. Res..

[28] Holzer RE, Slavin JA (1982). A quantitative model of geomagnetic activity. J. Geophys. Res..

[29] de La Sayette P, Berthelier A (1996). The am annual-diurnal variations 1959–1988: A 30-year evaluation. J. Geophys. Res..

[30] de La Sayette, P. Empirical simulations for the am annual-diurnal activity. *J. Geophys. Res. (Space Physics)* **109**, A07207. 10.1029/2003JA010353 (2004).

[31] Smith CW (1998). The ACE magnetic fields experiment. Sp. Sci. Rev..

[32] Stone E (1998). The advanced composition explorer. Sp. Sci. Rev..

[33] Crooker NU, Siscoe GL (1986). The effect of the solar wind on the terrestrial environment. Phys. Sun.

[34] Cliver EW, Kamide Y, Ling AG, Yokoyama N (2001). Semiannual variation of the geomagnetic Dst index: Evidence for a dominant nonstorm component. J. Geophys. Res..

[35] Lockwood, M. *et al.* Semi-annual, annual and Universal Time variations in the magnetosphere and in geomagnetic activity: 2. Response to solar wind power input and relationships with solar wind dynamic pressure and magnetospheric flux transport. *J. Sp. Weather Sp. Clim.* **10**, 30. 10.1051/swsc/2020033 (2020).

[36] Nitti, S. *et al.* Geomagnetic storm forecasting from solar coronal holes. *Mon. Notices R. Astron. Soc.* **519**, 3182–3193. 10.1093/mnras/stac3533. arXiv:2211.16572 (2023).

[37] Lockwood, M. & McWilliams, K. A. On optimum solar wind-magnetosphere coupling functions for transpolar voltage and planetary geomagnetic activity. *J. Geophys. Res. (Sp. Phys.)* **126**, e29946. 10.1029/2021JA029946 (2021).

